# Everyone wins

**DOI:** 10.7554/eLife.42676

**Published:** 2018-11-06

**Authors:** Renata C Matos, François Leulier

**Affiliations:** 1 Institut de Génomique Fonctionnelle de LyonUniversité Claude Bernard Lyon-1LyonFrance; 2 Institut de Génomique Fonctionnelle de LyonEcole Normale Supérieure de LyonLyonFrance

**Keywords:** inflammation, microbiome, lipocalin, Aeromonas, mutualism, AimA, Zebrafish

## Abstract

*Aeromonas* bacteria living in the gut of zebrafish produce a specific molecule to pacify the immune system of their host.

**Related research article** Rolig AS, Sweeney EG, Kaye LE, DeSantis MD, Perkins A, Banse AV, Hamilton MK, Guillemin K. 2018. A bacterial immunomodulatory protein with lipocalin-like domains facilitates host-bacteria mutualism in larval zebrafish. *eLife* **7**:e37172. doi: 10.7554/eLife.37172

Microbes can be found in almost every environment – including other organisms. Some cause disease, but many of them live with their hosts in a mutually beneficial relationship, a type of symbiosis called mutualism (McFall-Ngai et al., 2013). These microbial communities are in constant communication with the organisms hosting them, and gut bacteria in particular are known to influence the development, metabolism, immune system and behavior of their hosts (Fraune and Bosch, 2010; Kohl and Carey, 2016).

To peacefully cohabit with each other, the immune system of the host needs to control the number of bacteria without resorting to levels of inflammation that would harm either the bacteria or the host. As in many other areas of research, model organisms are widely used to dissect the molecular mechanisms that orchestrate such complex interactions. For example, the zebrafish – often used for studying development – is a powerful model that can be used to investigate such associations at the tissue, cell and molecular levels.

Most research to date has focused on how the host benefits from symbiosis, and the question of how the microbes benefit has been largely overlooked. Now, in eLife, Karen Guillemin and colleagues at the University of Oregon and the Canadian Institute for Advanced Research – including Annah Rolig as first author – report new details about the relationship between zebrafish larvae and one of their gut symbionts, *Aeromonas veronii* (Rolig et al., 2018).

*Aeromonas* are the only group of bacteria that are present throughout the zebrafish life cycle, and they play important roles in immune defense, gut cell growth and the development of the pancreas (Stephens et al., 2015; Burns and Guillemin, 2017). Rolig et al. used a specific strain of *A. veronii* (*Hm21*) to study the interactions between the bacteria and immune cells called neutrophils, which are the first immune cells to respond to inflammation and travel to the infected area (Brugman, 2016).

The fish were raised in sterile environments and were devoid of any microbes. Then, single bacteria were added and the interactions with the immune system were analyzed. This revealed that the bacteria produce a molecule that dampens the immune response of the neutrophils and so reduces inflammation.

The sequence of the molecule, which they named AimA (short for Aeromonas immune modulator A), did not resemble any known molecule, but its 3D crystal structure was similar to a molecule secreted by human neutrophils known as Lipocalin-2. Indeed, in experiments mimicking injuries to the zebrafish gut, the protective effect of AimA against inflammation was compromised when mouse Lipocalin-2 was added to the mix. This suggests that both molecules compete for the same receptors on the immune cells because they have similar structures.

Next, Rolig et al. investigated how AimA affected the ‘fitness’ of the *Aeromonas* and the zebrafish. The researchers created bacteria that lacked AimA and its close paralog AimB, and evaluated their ability to colonize larval zebrafish. Without AimA and AimB, the bacteria were unable to colonize the gut and the number of neutrophils increased, which in turn lead to more inflammation in the gut. However, when purified AimA was added, the mutant bacteria were able to accumulate in the gut and the number of neutrophils went back to normal.

This suggests that AimA mediates the interactions between zebrafish and the *Aeromonas* bacteria for the benefit of both partners, illustrating a prototypical example of mutualism. AimA is a colonization factor that intervenes early in the establishment of the bacteria in the gut, suppressing the immune response and inflammation in the gut to ensure the survival of the bacteria and the host. By doing so, it potentially allows bacteria to produce different molecules, which may benefit the host in other ways.

Symbiotic relationships have largely been described with a host-centric view, hence many molecular mechanisms may have been overlooked. Rolig et al. add AimA to the sparse list of known mutualistic factors, such as a specific polysaccharide that allows the bacterium *Bacteroides fragilis* to colonize the gut by activating tolerance in specific immune cells (Round et al., 2011). The anti-inflammatory potential of AimA and its atomic structure pave the way for researchers to test its potential as a therapeutic agent. The study of Rolig et al. highlights the importance of unbiased basic research in the host-microbe field using simple animal models.
